# Measuring the perceptual grouping of non-adjacent surfaces that are invisibly (amodally) or visibly connected

**DOI:** 10.1371/journal.pone.0208000

**Published:** 2018-11-28

**Authors:** Debarshi Datta, Howard S. Hock

**Affiliations:** 1 Department of Psychology, Florida Atlantic University, Boca Raton, Florida, United States of America; 2 Center for Complex Systems and Brain Sciences, Florida Atlantic University, Boca Raton, Florida, United States of America; University of Melbourne, AUSTRALIA

## Abstract

Classic Gestalt examples of perceptual grouping entail arrays of *disconnected* surfaces that are grouped on the basis of the surfaces’ relative similarity or proximity. However, most natural environments contain multiple objects, each with multiple, *connected* surfaces. Moreover, an object in a scene is likely to partially occlude other objects in the 2-dimensional retinal projection of the scene. A central question, therefore, is how the visual system forms a 3-dimensional representation of multi-object scenes by determining which surfaces belong to which objects. To this end, a recently developed *dynamic grouping* methodology determines whether pairs of surfaces are grouped together on the basis of the direction in which motion is perceived across a surface when its luminance is perturbed. It is shown using this method that the visible surfaces of a partially occluded object are perceptually grouped when they are plausibly connected and represented in a depth plane behind the occluding object. Invisible connectivity (amodal completion) as well as connectivity established by a visible surface have a powerful influence on the grouping of surfaces. However, for neither kind of connectivity is grouping affected by the distance between the surfaces. This absence of a distance/proximity effect on grouping is obtained when the space between to-be-grouped surfaces is filled with other surfaces. It contrasts with the strong effect of distance/proximity on the grouping of disconnected surfaces, and on the clarity of illusory contours formed between disconnected contours. It is concluded that distance/proximity is an operative grouping variable only when there is empty space between the to-be-grouped surfaces.

## Introduction

One of the primary challenges faced by the visual system is to construct a 3-dimensional representation of the visual world from the 2-dimensional projection of visual information onto our retinas. An important aspect of this challenge stems from the fact that objects in the world that are relatively close to the perceiver often are interposed between the perceiver and objects that are further away. As a result, the more distant objects are partially hidden behind the closer ones in the 2-dimensional retinal projection. The visual system’s incorporation of such hidden visual information into its 3-dimensional representation of the objects in a scene is called amodal completion [1]. Previous studies have been concerned with the stimulus conditions necessary for amodal completion, including good continuation [2], luminance similarity [3], and shape similarity [4]. Other researchers have shown that amodal completion can be affected by top-down influences entailing knowledge of the partially occluded objects [5,6].

The experiments reported in this article use a novel *dynamic grouping* methodology to determine whether amodal completion for a partially occluded object, like grouping via an intermediate connecting surface [7], is fundamentally a matter of perceptual organization (or unit formation [8]). In addition, it was determined whether the distance between surfaces affects their perceptual grouping when the surfaces are connected, either visibly by an intermediate surface, or amodally for a partially occluded object.

The dynamic grouping (DG) methodology, and the quantitative analysis based on it, have been developed in order to meet a particular theoretical need [9–11]. That is, although the contributions of the Gestalt psychologists [12] to our understanding of perceptual grouping cannot be over-estimated, most of these contributions entail perceptual grouping for arrays of disconnected surfaces. (See [13] for more recent advances using arrays of disconnected surfaces). The creation of the new methodology was motivated by the fact that both natural and manufactured objects are composed of connected rather than disconnected surfaces. In order to understand object perception, it was deemed necessary to determine how the *connected* surfaces of an object are grouped together; i.e., the compositional structure of the object. It has been shown with the DG methodology that intuitive Gestalt grouping variables (good continuation) and less intuitive grouping variables (common luminance polarity [14]) affect the perceptual grouping of connected surfaces [9]).

Previous quantitative analyses of perceptual grouping have focused on specific factors that affect amodal completion. For example, a measure of relatability for discontinuous contours has been developed [8] that depends on how well they can be connected by interpolated monotonic contours (i.e., without inflection points [15]). Subsequent studies [16,17] have determined how precisely observers can localize such interpolated contours. These investigations are directly relevant to the alignment/good-continuation of disconnected surfaces, a major determinant of amodal completion [2,18]. In contrast, the DG methodology is not specific to a particular measure; It integrates the effects of grouping variables besides alignment/relatability in determining the likelihood that the surfaces will be grouped.

**Fig 1:**
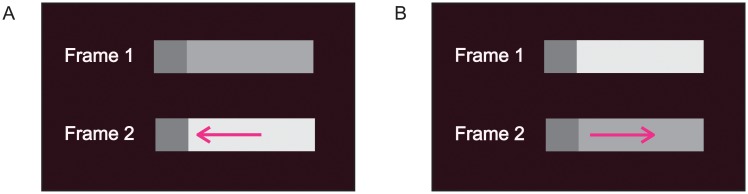
The direction of dynamic grouping motion. As indicated by arrows, perceived dynamic grouping motion across a surface whose luminance is perturbed is in a direction that indicates whether there has been a decrease in the similarity (affinity) of the connected surfaces (A), or whether there has been an increase in their similarity (affinity) (B). (From [9]).

The dynamic grouping methodology determines whether two connected surfaces are grouped on the basis of the perception of motion. Dynamic grouping motion is induced when an attribute that is shared by two connected surfaces is changed for one of the surfaces. It then appears as if the new value of the attribute were painted across the surface. When the perturbed surface’s grouping strength (affinity) with the unchanged, connected surface has decreased (when they become less similar), perceived DG motion across the perturbed surface is toward the boundary of the surfaces, as in Fig 1A. When the perturbed surface’s grouping strength (affinity) with the unchanged connected surface has increased (when they become more similar), perceived DG motion across the perturbed surface is away from the boundary of the surfaces, as in Fig 1B.

Grouping variables combine cooperatively and nonlinearly (super-additively) in determining the pre-perturbation affinity of connected surfaces [11], and the strength of the DG motion is determined by how much their affinity changes when a dynamic grouping variable is perturbed [9]. When it is possible for a perturbed surface to be grouped with one or the other of two surfaces with which it is connected, the direction of DG motion indicates the surface with which the perturbed surface had the greater affinity prior to the perturbation.

The experiments reported in this article investigate characteristics of amodal completion that reflect the 3-dimensional representation of occluding and partially occluded objects. These are characteristics that are not literally present in their 2-dimensional retinal projection. It was determined in Experiments 1 and 2 whether: 1) non-adjacent, visible surfaces of a partially occluded object are perceptually grouped only when they are aligned/relatable, and therefore plausibly connected, and 2) the perceptually grouped surfaces of a partially occluded object are segregated from the occluding object by being represented in a depth plane behind the occluding object. In Experiment 3 it was determined whether the amodal connection of non-adjacent surfaces behind an occluding object, although invisible, results in surface grouping that is similar in strength to non-adjacent surfaces that are visibly connected by an intermediate surface (the latter differs in luminance from the surfaces it connects, as per ‘*element* connectedness’ [7]. Finally, it was determined in Experiment 4 whether distance/proximity, which strongly affects perceptual grouping when surfaces are disconnected, also affects their grouping when they are either invisibly (amodally) or visibly connected.

## General method

**Fig 2:**
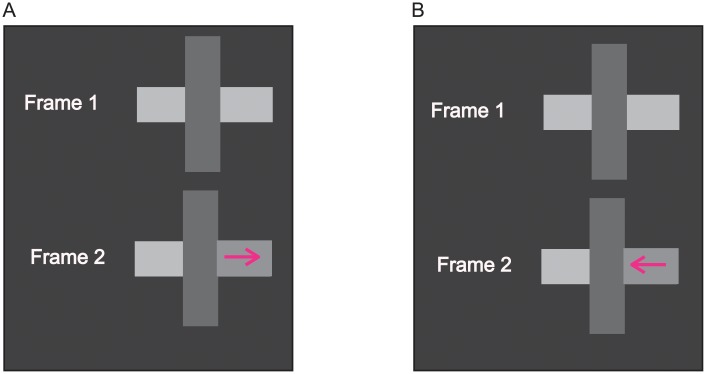
Experimental paradigm for investigating amodal completion. A luminance decrement for one of the horizontal surfaces results in it becoming more similar to the vertical surface, so if horizontal-vertical were the preferred grouping, dynamic grouping motion would be perceived across the decremented surface in the rightward direction (A). A luminance decrement for one of the horizontal surfaces also results in it becoming less similar to the opposite horizontal surface, so if horizontal-horizontal were the preferred grouping, dynamic grouping motion would be perceived across the decremented surface in the leftward direction (B). The Frame 2 luminance changes depicted in the figure are an inexact illustration of an easily detected luminance decrement.

The application of the dynamic grouping methodology to the investigation of amodal completion is illustrated in Fig 2 for a geometric stimulus composed of three surfaces. For this stimulus, there appears to be amodal completion of a horizontally oriented surface behind a darker, vertically oriented occluding surface. DG motion is perceived across one of the two visible horizontal surfaces (the randomly determined target for a trial) when its luminance is decreased. The luminance decrement increases the perturbed surface’s similarity with the adjacent vertical surface, so if this were the preferred surface grouping, DG motion would be perceived across the decremented horizontal surface, away from its boundary with the vertical surface. The horizontal-vertical grouping is indicated by rightward motion in Fig 2A. However, the luminance decrement also decreases the similarity of the decremented surface with the horizontal surface on the other side of the vertical surface. If this were the preferred surface grouping, perceived DG motion would be perceived across the decremented surface, toward the other horizontal surface. The horizontal-horizontal grouping is indicated by leftward motion in Fig 2B. This is the surface grouping that would be expected if a horizontally oriented object were amodally completed behind the vertically oriented occluding object.

### Stimuli

Stimuli were presented on the darkened screen of a Mitsubishi Diamond Pro 930 ^sb^ monitor. Screen luminance < 0.01 cd/m^2^. The screen size was 356 x 266 centimeters (cm) and **t**he pixel size was 0.035 cm. Each pixel intercepted a visual angle of 0.7 degrees (deg) when viewed from a distance of 30 cm, which was maintained with a head restraint. Stimulus presentation and the recording of responses were controlled by MATLAB scripts in conjunction with the Psychophysical Toolbox-Version 3 (PTB-3) [19–21].

Each stimulus was composed of three surfaces: two horizontally oriented surfaces on either side of a central surface. In each experiment the size of both horizontal surfaces was 5.52 x 1.38 degrees (deg) of visual angle, and their luminance during the first frame of each trial was 56.5 candelas per square meter (cd/m^2^). The luminance of the central surface remained at 5.9 cd/m^2^ in each experiment. In Experiment 1 the central surface was a vertical rectangle (3.26 x 8.28 deg), and the horizontal surfaces were either aligned or mis-aligned. In Experiment 2 the central surface was a vertical rectangle (3.26 x 8.28 deg) that was stereoscopically in front of or behind the horizontal surfaces. In Experiment 3 the central surface was always 3.86 deg wide. It either was 8.28, 0.69, or 0.14 deg high. In Experiment 4 the central surface was either 8.28 deg or 0.14 deg high. Its width was 0.83, 3.86, or 6.90 deg.

Each trial began with the 2000 millisecond (msec) presentation of a fixation dot in the center of one or the other of the to-be-presented horizontal surfaces. This was the randomly-determined target surface for that trial. The fixation dot disappeared upon the presentation of the 3-surface stimulus during the 500 msec first frame. During the 500 msec second frame, the luminance of the target horizontal surface was decremented by 3.5, 4.6, 5.5, 6.5, 7.7, 8.7, or 11.0 cd/m^2^. The inter-frame interval was 0 msec.

The luminance decrements for the target surface decreased edge contrast at its boundary with the central surface, and also decreased the horizontal surface’s contrast with the background. Thus, there were no contrast increases (luminance increments) to combine with the contrast decreases (luminance decrements). The multiplicative combination of such oppositely signed contrast changes would have resulted in the perception of a counterchange-specified motion. The counterchange motion would have started at the decrease in contrast and ended at the increase in contrast [22,23]. It was eliminated as a possibility in order for the perceived motion to be solely indicative of the dynamic grouping of connected surfaces.

### Design and procedure

Following several practice sessions, there were 4 blocks of trials per testing session (Experiments 1 and 2) or 3 blocks of trials per testing session (Experiments 3 and 4). The blocks of trials differed with respect to the nature of the 3-surface stimulus. Their order was counterbalanced over 2 sessions (Experiments 1 and 2) or 6 sessions (Experiments 3 and 4). Each block comprised 70 order-randomized trials determined by the orthogonal combination of 2 target locations (the left- or right-hand horizontal surface), 7 luminance decrements for the target horizontal surface, and 5 repetitions.

After each trial participants pressed keys on the computer keyboard to indicate whether or not they perceived motion, and if so, whether it was leftward, rightward, or in both directions at the same time (i.e., bidirectional). Instead of these responses, they were instructed to press the space bar when they were unsure of the motion direction during that trial or had momentarily lost attention.

### Participants

There were eleven voluntary participants, four in each of four experiments. One participated in Experiments 1 and 2, and four participated in Experiments 3 and 4. Their sex and age are presented within each experiment. All were students at Florida Atlantic University who were naive with respect to the purposes of the experiments.

All participants provided signed informed consent that was approved by the Florida Atlantic University Institutional Review Board (IRB), which also exempted this study from specific ethical approval (H03-195).

### Analysis of the results

**I**n all four experiments, the perception of ‘no motion’ predominated when the frame-2 luminance decrements were small. The perception of DG leftward or rightward motion across the target horizontal surface increased as the size of the luminance decrement increased. Space-bar responses indicating uncertainty or loss of attention occurred for 0.5% of the trials in Experiment 1, 0.1% in Experiment 2, 9.1% in Experiment 3 and 13.3% in Experiment 4. Because mean proportions were very variable when they were based on only a few trials, they were not computed for a luminance decrement when the space bar was pressed for more than half of a participant’s responses. This occurred for 0.8% of the trials in Experiment 3 and 0.2% of the trials in Experiment 4.

The proportion of trials indicating that the two horizontal surfaces were perceptually grouped was determined by averaging, for each luminance decrement, the proportion of trials for which leftward DG motion was perceived when the horizontal surface on the right was the target, with the proportion of trials for which rightward DG motion was perceived when the horizontal surface on the left was the target. The proportion of trials indicating that the target horizontal surface was grouped with the adjacent central surface was determined by averaging, for each luminance decrement, the proportion of trials for which rightward DG motion was perceived when the horizontal surface on the right was the target, with the proportion of trials for which leftward DG motion was perceived when the horizontal surface on the left was the target. See S1 Dataset for individual participant’s results in each experiment.

### Statistics

All the statistical results entailed the use of SSPS 25 to conduct analyses of variance (ANOVAs) with repeated measures. In Experiment 1 the dependent variable was the proportion of trials that motion was perceived (in a direction indicating either horizontal-horizontal or horizontal-vertical grouping). In Experiment 2 the dependent variable was the proportion of trials that motion was perceived in a direction indicative of horizontal-horizontal grouping in one ANOVA, and the proportion of trials that motion was not perceived in another ANOVA. In Experiments 3 and 4 the dependent variable was the proportion of trials that motion was perceived in a direction indicative of horizontal-horizontal grouping.

Tests of sphericity [24] were not significant for any of the experiments, so sphericity was assumed for all the ANOVAs. Effect size, partial-eta squared (η^2^) and a measure of power are included with the statistical results for each experiment.

## Experiment 1

The objective of this experiment was to determine whether the perceived DG motion direction is consistent with the grouping of the visible surfaces of a partially occluded object, and to then determine whether their grouping depended on whether the surfaces were plausibly connected behind an occluding surface.

**Fig 3:**
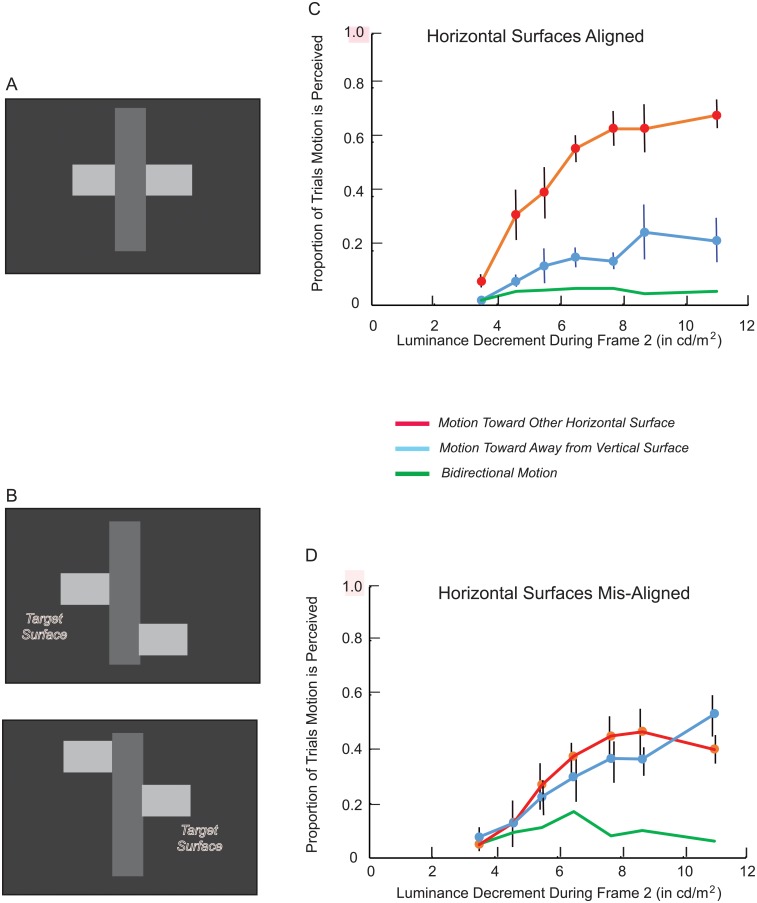
Experiment 1: Stimuli and results. Stimuli: Horizontal surfaces are aligned (A) or mis-aligned (B). When mis-aligned, the target horizontal surface always was centered with respect to the vertical surface. illustrative demos are included with the Supplementary Information (S1 and S2 Movies). Results: In the aligned (C) and mis-aligned (D) conditions, the proportion of trials motion was perceived in a direction indicating that the decremented horizontal surface was grouped with either the other horizontal surface, or with the central, vertical surface. Included in panels C and D are the proportions of trials ‘bidirectional’ motion was perceived. Vertical lines in panels C and D indicate +/- one standard error. Standard errors smaller than the markers are omitted.

The horizontally oriented surfaces were connected to an intermediate, vertically oriented surface in the 2-dimensional retinal projection of the stimulus, regardless of whether they were aligned or mis-aligned. However, only when they were aligned were the horizontal surfaces plausibly connected in the 3-dimensional representation of the stimulus. It was anticipated that when the luminance decrement of the target horizontal surface was sufficiently large, DG motion would be perceived. It would be toward the other horizontal surface more often when they were aligned, and therefore plausibly connected (Fig 3A) compared with when they were mis-aligned (Fig 3B). The height of the vertical surface, 8.28 deg, exceeded the height of the horizontal surfaces (1.38 deg), so it could be perceived as an occluder of a horizontally oriented object behind it. When the horizontal surfaces were mis-aligned, the vertical gap between them was 0.7 deg, the target horizontal surface always was centered with respect to the vertical surface, and the non-target horizontal surface was shifted downward or upward (Fig 3B). Blocks of trials in which the horizontal surfaces were aligned were alternated with blocks of trials in which the horizontal surfaces were mis-aligned (two of each per session). For half the participants, the aligned blocks preceded the mis-aligned blocks during the first testing session, and the order was reversed during the second testing session. These orders were inverted for the other half of the participants. All four participants, P1, P2, P3 and P4 were female. Their ages were 23, 19, 18 and 23, respectively.

### Results

When the horizontal surfaces were aligned it was plausible for them to be connected behind the vertical surface. DG motion was perceived in a direction indicative of horizontal-horizontal grouping more often than it was perceived in a direction indicative of horizontal-vertical grouping (Fig 3C). Analysis of variance indicated that the difference between the two directions of DG motion was statistically significant (F(1,3) = 13.43; p < .05; η^2^ = 0.82; power = 0.67). The perceptual grouping specified by the direction of DG motion therefore was consistent with what would be expected for the amodal completion of the visible parts of a horizontally oriented object behind an occluding object. Although the DG methodology was developed in order to account for the grouping of visibly connected, adjacent surfaces, the results for the ‘aligned’ condition indicate that it could account as well for the grouping of non-adjacent, but amodally connected surfaces.

Amodal completion was not indicated when the horizontal surfaces were mis-aligned; DG motion in a direction indicative of horizontal-vertical grouping was perceived approximately equally often as DG motions in a direction indicative of horizontal-horizontal grouping (Fig 3D). Analysis of variance indicated that the difference between the these opposing DG motion directions was not statistically significant (F(1,3) = 0.13, p > .05; η^2^ = 0.0.04; power = 0.06).

## Experiment 2

Amodal completion implies that pairs of surfaces are invisibly connected in the 3-dimensional representation of a scene. However, the stimuli illustrated in Fig 2A are visibly connected in the 2-dimensional plane. This “element connectivity” [7] might have been the basis for the perceptual grouping of the horizontal surfaces in the ‘aligned’ condition of Experiment 1.

**Fig 4:**
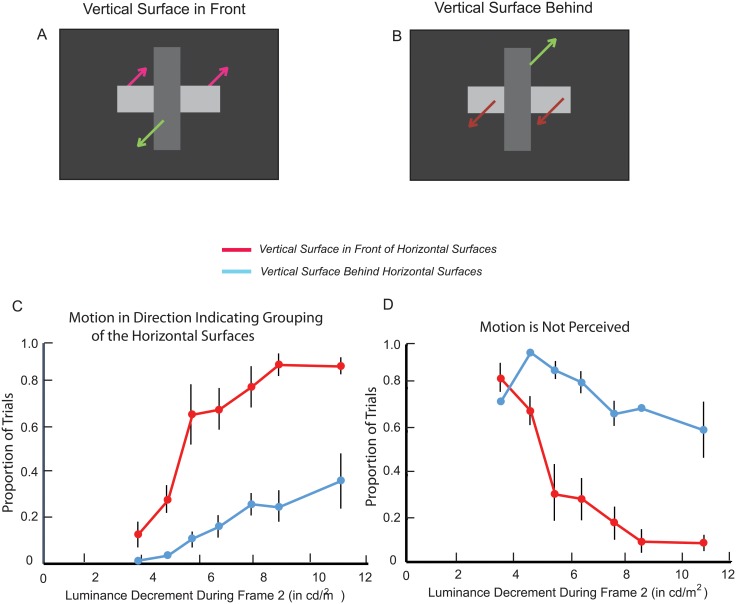
Experiment 2: Stimuli and results. Stimuli: The vertical surface is stereoscopically in front of the horizontal surfaces (A) or behind them (B). The green arrow indicates whether the vertical surface is stereoscopically “placed” forward or backward. The same is indicated for the horizontal surfaces by the red arrows. Results: The proportion of trials motion was perceived in a direction indicating that the decremented horizontal surface was grouped with the other horizontal surface (C), and trials for which motion was not perceived (D). There were too few ‘bidirectional’ responses, and too few responses in directions indicative of horizontal-vertical grouping to include in the graph. Vertical lines in C and D indicate +/- one standard error. Standard errors smaller than the markers are omitted.

This possibility was addressed in Experiment 2 by stereoscopically presenting the vertical and horizontal surfaces in different depth planes. Regardless of whether the vertical surface was stereoscopically in front of or behind the horizontal surfaces, the horizontal surfaces were never visibly connected to each other or to the intermediate vertical surface. It could be determined, therefore, whether the amodal completion of a partially occluded object does indeed entail the formation of a 3-dimensional representation. When the horizontal surfaces were perceptually segregated from the vertical surface by being stereoscopically behind it (Fig 4A), it was possible for the horizontal surfaces to be invisibly (amodally) connected. DG motion in a direction indicative of horizontal-horizontal grouping was anticipated. When the horizontal surfaces were perceptually segregated from the vertical surface by being stereoscopically in front of it (Fig 4B), the horizontal surfaces were not visibly connected, and it was implausible for them to be invisibly (amodally) connected. Relatively little DG motion perception was anticipated.

### Method

The mirrors of an Optosigma 4-Mirror stereoscope were adjusted by the participants prior to the start of each testing session in order to confirm that the stereoscopic difference in depth between the horizontal and vertical surfaces was maintained when the target horizontal surface changed randomly from one trial to the next. The sizes and luminance values of the horizontal and vertical surfaces were as in Experiment 1. Blocks of trials in which the horizontal surfaces were either behind or in front of the vertical surface were alternated (two of each per session). For half the participants, the “horizontal behind” blocks preceded the “horizontal in front” blocks for the first testing session, and their order was reversed during the second testing session. These orders were inverted for the other half of the participants. The four participants were: P1-female, age23; P5-male, age 22, P6-female, age 31, and P7-male, age 27.

### Results

DG motion was perceived in a direction indicative of the grouping of the horizontal surfaces more often when they were stereoscopically behind the vertical surface compared with when they were stereoscopically in front of the vertical surface (Fig 4C). Analysis of variance indicated that this difference in DG motion perception was statistically significant (F(1,3) = 29.22, p < .02; η^2^ = 0.91; power = 0.93). This was consistent with the amodal completion of a horizontally oriented object behind a vertically oriented occluding object.

The perception of ‘no motion’ rather than DG motion predominated when the horizontal surfaces were stereoscopically in front of the vertical surface (Fig 4D). Analysis of variance indicated that ‘no motion’ was reported significantly more often when the horizontal surfaces were stereoscopically in front compared with when they were stereoscopically behind the vertical surface (F(1,3) = 29.81, p < .02; η^2^ = 0.91; power = 0.94). Also statistically significant in the ANOVA for ‘no motion’ responses was the interaction between the relative depth of the horizontal and vertical surfaces and the size of the luminance decrement (F(1,6) = 8.50, p < .001; η^2^ = 0.74; power = 0.94). When the horizontal surfaces were behind the vertical surface, ‘no motion’ responses decreased as the size of the luminance decrement increased, as in the other experiments reported in this article. However, when the horizontal surfaces were in front of the vertical surface, ‘no motion’ responses frequently occurred even for large luminance decrements. Nonetheless, DG motion across the target horizontal surface, when perceived, was in a direction indicative of grouping with the other horizontal surface. This suggested that the DG methodology might reveal aspects of perceptual organization for surfaces that are neither visibly nor invisibly connected.

## Experiment 3

**Fig 5:**
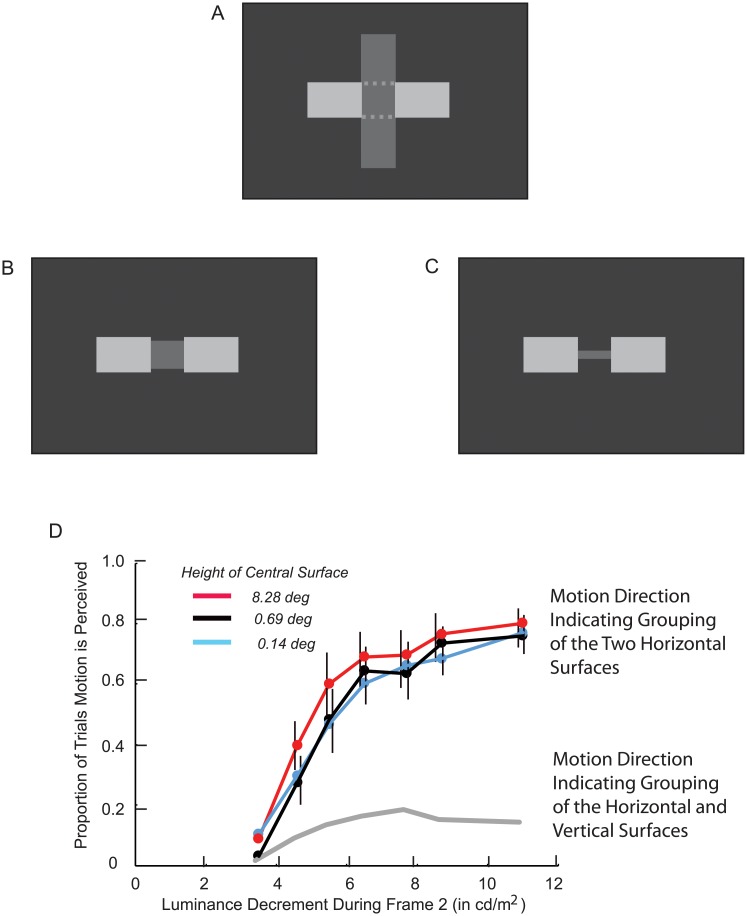
Experiment 3: Stimuli and results. Stimuli: Horizontal surfaces are connected by a central surface that can function as an occluder (A) or can be too short to function as an occluder and can only function as a visible connector (B and C). Results: Proportion of trials motion is perceived in a direction indicating that the decremented horizontal surface is grouped with the other horizontal surface (D). Included in panel D are the proportions of trials motion was perceived in a direction indicating that the decremented horizontal surface was grouped with the central surface (averaged for the three heights of the central surface). There were too few ‘bidirectional’ responses to include in the graph. Vertical lines in panel D indicate +/- one standard error. The standard error bars are sometimes displaced so that they don’t over-lap for different central surface heights. Standard errors smaller than the markers are omitted. To minimize clutter that would reduce legibility, standard errors are averaged over the two non-occluder conditions.

Evidence for horizontal-horizontal grouping was obtained in Experiment 2 only when the horizontal surfaces were stereoscopically behind the vertical surface. This ruled out the possibility that the horizontal-horizontal grouping was due to connectivity via an intermediate surface (i.e., ‘element connectivity’). It was determined in this experiment whether the perception of DG motion is comparable for horizontal surfaces whose connection is amodal (Fig 5A) and horizontal surfaces that are visibly connected by intermediate surfaces that are not plausible occluders (Fig 5B and 5C).

There were three blocks of trials, one for each of the three heights of the central surface (8.28. 0.69 and 0.14 deg). The height of the horizontal surfaces was 1.38 deg, so occlusion was plausible only when the height of the central surface was 8.28 deg. Each of the blocks was tested once per testing session. Their order was counterbalanced over six testing sessions. As in the preceding experiments, the width of the central surface was 3.86 deg. The four participants were: P8-male, age 22; P9-female, age 25, P10-female, age 56, and P11-male, age 24.

### Results

The perception of DG motion was predominantly in a direction consistent with the grouping of the two horizontal surfaces, regardless of whether the central surface was a plausible occluder, or whether it could only function as a visible connector (Fig 5D). It can be seen in the figure that there was considerable variability among the participants in how often DG motion was perceived. Nonetheless, the effect of the central surface’s height was sufficiently consistent for each participant for the ANOVA to be marginally significant (F(2,6) = 4.84, p = .056; η^2^ = 0.62; power = 0.57). Marginal statistical significance aside, there was at best a small difference in horizontal-horizontal grouping when their connection was invisible (amodal) compared with when it was visible.

## Experiment 4

It has previously been shown [7] that the grouping of surfaces does not depend on the distance between them when the intermediate surface connecting them has the same luminance as the to-be-grouped surfaces (‘uniform connectivity). They also have shown that the grouping of surfaces does not depend on the similarity of their shapes when the intermediate surface connecting them differs in luminance from the to-be-grouped surfaces (‘element connectivity). The current experiment determined whether invisible (amodal) connectivity and visible (element) connectivity are like uniform connectivity in that the grouping of surfaces does not depend on the distance between them.

**Fig 6:**
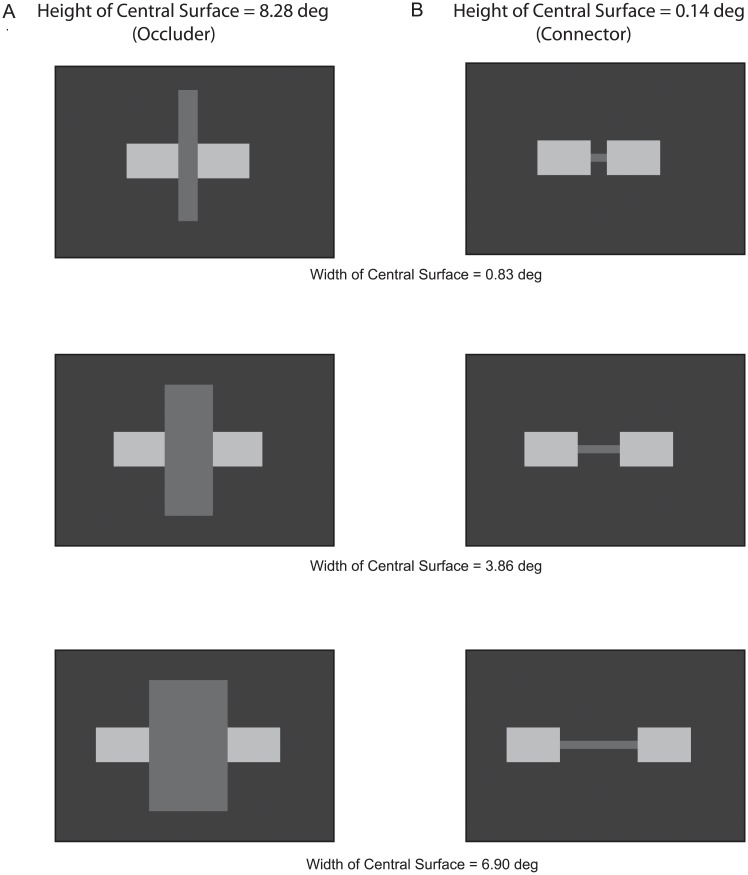
Experiment 4: Stimuli. Horizontal surfaces are connected by a central surface of varying width that could function as an occluder (A) or is too short to function as an occluder and could only function as a visible connector (B).

The prediction based on results for connected surfaces [7] was that there would be no effect of distance on the perception of DG motion in the direction indicative of horizontal-horizontal grouping. This prediction contrasts with the effect of distance/proximity that is typically observed for arrays of disconnected surfaces [12] and disconnected contours [25,8]. For both invisible connectivity (Fig 6A) and visible connectivity (Fig 6B), the effects of distance were tested by varying the width of the central surface between the two horizontal surfaces.

The spatial dimensions of the horizontal surfaces were as in the preceding experiments, and the width of the central surface was 0.83, 3.86, or 6.90 deg. In the first part of the experiment, the height of the central surface was 8.28 deg (Fig 6A), so it could function as an occluder. In the second part of the experiment, the height of the central surface was 0.14 deg (Fig 6B), so it only could function as a connector. There were three blocks of trials during each part of the experiment, one for each of the three widths of the central surface. Their order was counterbalanced over six testing sessions. The four participants were: P8-male, age 22; P9-female, age 25, P10-female, age 56, and P11-male, age 24.

### Results

**Fig 7:**
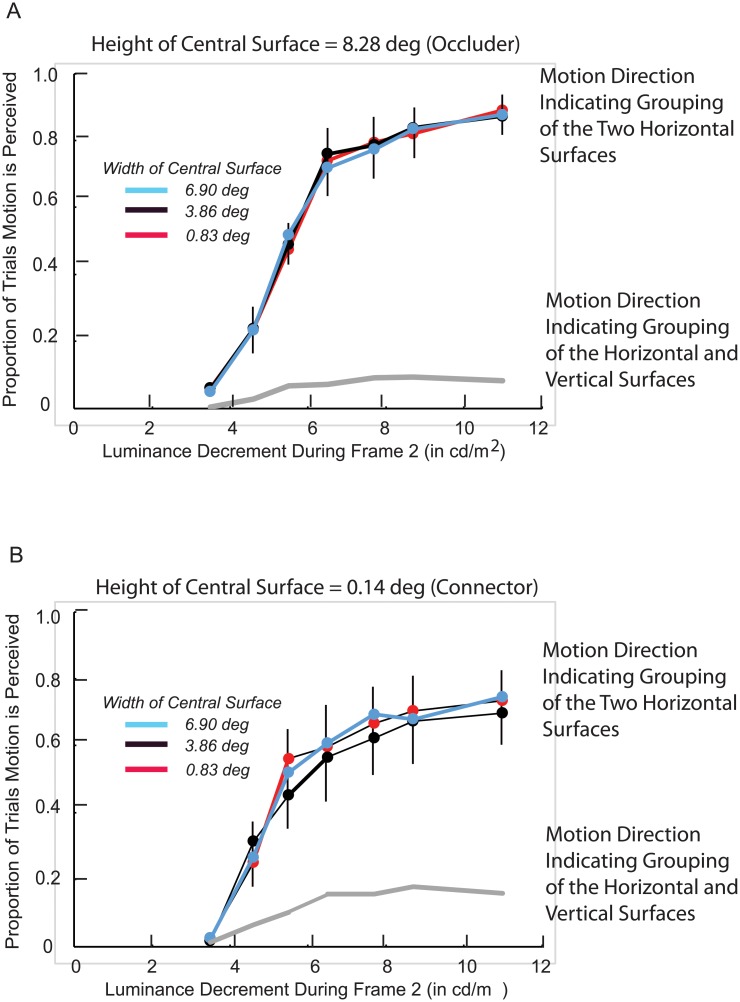
Experiment 4: Results. Proportion of trials motion is perceived in a direction indicating that the target horizontal surface was grouped with the other horizontal surface. This is graphed separately when the central surface could function as an occluder (A) and when it could only function as a visible connector (B). Included in panels A and B are the proportions of trials motion was perceived in a direction indicating that the decremented horizontal surface was grouped with the vertical surface. There were too few ‘bidirectional’ responses to include in the graph. Vertical lines indicate +/- one standard error. The standard error bars are sometimes displaced so that they don’t over-lap for different central surface heights. They are omitted when smaller than the markers. To minimize clutter that would reduce legibility, standard errors are averaged over the three widths of the central surface.

The proportions of trials that DG motion was perceived in a direction that was consistent with the grouping of the two horizontal surfaces are presented in Fig 7. ANOVAs for both the ‘occluder’ and ‘visible connector’ conditions indicated that there was little if any effect of the distance between the horizontal surfaces on DG motion indicating horizontal-horizontal grouping. This was the case for the ‘invisible connector’ (amodal) condition (F(2,6) = 0.13, p > .05; η^2^ = 0.04; power = 0.06) and for the ‘visible connector’ condition (F(2,6) = 0.9, p > .05; η^2^ = 0.24; power = 0.15). Given the prediction of a null effect of distance based on previously reported results [7], increasing the experiment’s power would not be expected to alter the outcome.

### Discussion

It has been has found [25] that the grouping of disconnected contours (as determined by whether they were perceived rotating in the same direction) depends on the ratio of the contours’ length to the size of the gap between them; larger ratios were correlated with stronger grouping. Similarly, it has been shown that the clarity of illusory contours depends on the length of the disconnected inducing contours relative to the gap between them [8,26].

Instead of the above, the results of Experiment 4 were consistent with evidence [7] that the distance between two surfaces would not affect their grouping when the surfaces are connected. The width of the horizontal surfaces was fixed at 5.52 deg, but the varying distance between them (0.83, 3.86, or 6.90 deg) did not affect their grouping (as determined by the perception of dynamic grouping motion). This was the case regardless of whether the connection between the horizontal surfaces was visible or invisible (amodal) in the 3-dimensional representation of the stimulus.

## General discussion

The experiments reported in the article provide evidence for the efficacy of a new method for studying perceptual organization: dynamic grouping (DG). The method, which was developed to investigate the grouping of connected, adjacent surfaces, has provided evidence in the current study for the grouping of invisibly (amodally) connected, non-adjacent surfaces, as well as surfaces that are visibly connected by an intermediate surface. Experiments 1 and 2 provided evidence for the perceptual grouping (amodal completion) of surfaces that are disconnected in 2-dimensional space by a partially occluding object. It was shown that this depended on the surfaces being plausibly connected in the 3-dimensional representation of a scene. The results of Experiment 3 indicated that the grouping of two surfaces via invisible connection (amodal completion) is at best marginally stronger than their grouping via a visible connecting surface. Finally, it was found in Experiment 4 that the distance between two surfaces did not affect their grouping, regardless of whether they were visibly or invisibly (amodally) connected.

### Competition between alternative surface groupings

In previous studies it has been shown that the pre-perturbation affinity of two surfaces depends on the super-additive combination of the effects of individual grouping variables 9–11]. Thus, discretely changing (perturbing) an attribute of a surface (e.g., its luminance in the current study) affects its similarity with, and therefore its affinity with other surfaces. When additional grouping variables contribute to the surfaces’ affinity, the same change in luminance similarity produces a larger change in affinity, and thereby, stronger DG motion.

**Fig 8:**
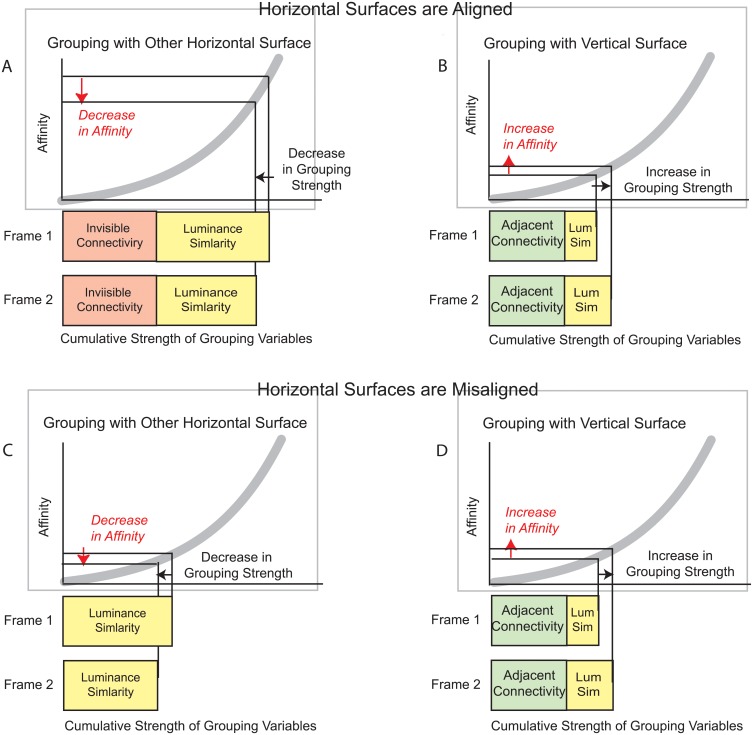
Surface affinity. Grouping variables combine nonlinearly (super-additively) to determine the over-all affinity for pairs of surfaces. Changes in a grouping variable, in this case luminance similarity, produce a change in affinity. The strength of the induced dynamic grouping (DG) motion depends on the size of the affinity change. This is illustrated for the ‘aligned’ (panels A and B) and the ‘mis-aligned’(panels C and D) conditions of Experiment 1. Note that affinity decreases for the horizontal-horizontal grouping (panels A and C) and affinity increases for the horizontal-vertical grouping (panels B and C), creating competing DG motions in opposite directions.

When alternative surface groupings are possible, the relative size of the affinity-changes for the alternative groupings determines the relative strengths of the competing DG motions. This is shown in Fig 8 for the aligned and mis-aligned conditions of Experiment 1. Both conditions entail competition between DG motion indicative of horizontal-horizontal grouping (Fig 8A and 8C), and DG motion indicative of the horizontal-vertical grouping (Fig 8B and 8D).

In the ‘aligned’ condition (Fig 8A and 8B) both of the competing surface groupings are affected by their connectivity; there is invisible connectivity for the horizontal-horizontal grouping and adjacent connectivity for the horizontal-vertical grouping. However, the horizontal-horizontal grouping has a substantial advantage due to pre-perturbation luminance similarity. That is, luminance was identical for the two horizontal surfaces prior to the perturbation, but the vertical surface was much darker than the horizontal surfaces. As a result of the greater pre-perturbation affinity of the horizontal-horizontal grouping, the same change in luminance similarity produces a much larger change in affinity for the horizontal-horizontal grouping than the horizontal-vertical grouping. This induced stronger DG motion in a direction that was consistent with horizontal-horizontal grouping compared with the DG motion in the opposing direction, which is indicative of horizontal-vertical grouping.

Invisible connectivity (amodal completion) was not plausible when the horizontal surfaces were mis-aligned (Figs 8C and 8D), so the pre-perturbation affinity of the horizontal surfaces was similar to the pre-perturbation affinity of the horizontal and vertical surfaces. As a result, the same change in affinity due to the luminance perturbation produced DG motions of similar strength, but in opposite directions. The alternative DG motion directions compete in the same way as in other forms of motion perception. For example, both horizontal and vertical motions are stimulated for the motion quartet, but because of inhibitory competition only one or the other is perceived [27]. Most often it is the stronger DG motion direction that is perceived, and the weaker alternative DG motion direction that is suppressed [11], as was observed in the ‘aligned’ condition of Experiment 1. In the ‘mis-aligned’ condition of Experiment 1, DG motion indicative of horizontal-horizontal and horizontal-vertical grouping were perceived approximately equally often. Which motion direction would be perceived and which would be suppressed would then be determined by random fluctuations in motion-detector activation.

### The effect of distance/proximity

It is well established that the distance between *dis*connected surfaces has a strong effect on their perceptual grouping [12,25,8]. However, consistent with previously reported results [7], no effect of distance on horizontal-horizontal grouping was observed in Experiment 4, regardless of whether the horizontal surfaces were invisibly (amodally) or visibly connected. Because there was a high level of pre-perturbation affinity for the horizontal surfaces due to their luminance similarity, it would be expected because of super-additivity that even a small effect of the distance between the horizontal surfaces would have a significant additional effect on affinity. In the absence of such an effect, it was concluded that there was no effect of the distance between the horizontal surfaces on their perceptual grouping. It might be argued, however, that the high levels of pre-perturbation affinity for the horizontal surfaces created a kind of ceiling effect that minimized the effect of distance. Although plausible, this possibility is contradicted by evidence from earlier studies indicating that high levels of pre-perturbation affinity due to connectivity and luminance similarity enhance rather than suppress the effects of other grouping variables on dynamic grouping motion [11]. Nor did high levels of pre-perturbation affinity for the horizontal surfaces “overwhelm” the effect of alignment in Experiment 1 of the current study.

Although many of the same variables that affect the grouping of disconnected surfaces also affect the grouping of connected surfaces, it is not necessary for this to be the case for all potential grouping variables. Distance/proximity may not affect the grouping of connected surfaces because the distance between any two surfaces is determined by the length of the empty space (the gap) between them. When the space between to-be-grouped surfaces is filled with other surfaces (as in our experiments and [7]), there is no distance to detect because the surfaces cannot be segregated by figure-ground processes. Only when surfaces (contours) are disconnected can they be segregated, and only then is there a discernible gap separating the surfaces.

In the absence of empty space between to-be-grouped surfaces, the distance between them is no longer an operative grouping variable. As such, it would not be expected to contribute to the grouping strength/affinity of connected surfaces. (Analogously, hue similarity would have no influence on the grouping of achromatic surfaces because the hue of the surfaces is not determined).

## Conclusion

The results of this study provide further evidence for the usefulness of the dynamic grouping (DG) method for determining the perceptual grouping of connected surfaces. It was shown that the effect of connectivity is not limited to adjacency. Non-adjacent surfaces can be perceptually grouped if they were invisibly connected behind an occluder or visibly connected by an intermediate surface. The results therefore contribute to our understanding of how the visual system forms 3-dimensional representations for multi-object scenes. How is it determined which surfaces in the scene belong to the same object, and which belong to different objects? Early computational approaches [28,29] have addressed this question by distinguishing between different kinds of intersections formed by the boundaries of the surfaces while ignoring the other attributes of the surfaces. In contrast, dynamic grouping empirically assesses surface affinities, as determined by all the shared attributes of to-be-grouped surfaces. Both surfaces and their boundaries are of obvious importance, so a theoretical approach that combines the analysis of boundary intersections with the determination of surface affinities might provide a significant advance in our understanding of the 3-dimensional representation of complex scenes.

## Supporting information

S1 Dataset Supplementary data. (XLSX)

S1 Movie Aligned. (MOV)

S2 Movie Mis-aligned. (MOV)
